# Intra-Abdominal Desmoplastic Small Round Cell Tumor (DSRCT) and the Role of Hyperthermic Intraperitoneal Chemotherapy (HIPEC): A Review

**DOI:** 10.3390/curroncol30040299

**Published:** 2023-03-31

**Authors:** Sophie J. M. Reijers, Caroline C. H. Siew, Niels F. M. Kok, Charles Honoré, Winan J. van Houdt

**Affiliations:** 1 Department of Surgical Oncology, Netherlands Cancer Institute, 1066 Amsterdam, The Netherlands; s.reijers@nki.nl (S.J.M.R.);; 2 Department of General Surgery, Tan Tock Seng Hospital, Singapore 308433, Singapore; 3 Department of Surgery, Gustave Roussy Cancer Center, 94805 Villejuif, France

**Keywords:** DSRCT, desmoplastic small round cell tumor, CRS, cytoreductive surgery, HIPEC, hyperthermic intraperitoneal chemotherapy

## Abstract

Desmoplastic small round cell tumor is a very rare and highly aggressive soft tissue sarcoma, usually presenting with multiple intra-abdominal tumors in young males. Patients present with advanced disease and the overall survival is dismal. Multiple studies report relatively favorable outcomes with multimodal treatment consisting of chemotherapy, surgery and radiotherapy. If resection is feasible, complete cytoreductive surgery is the cornerstone of surgical treatment. The benefit of hyperthermic intraperitoneal chemotherapy in addition to cytoreductive surgery is unclear, and few studies have evaluated this option. We sought to identify the role of hyperthermic intraperitoneal chemotherapy in patients with intra-abdominal desmoplastic small round cell tumor. Our review of the available literature revealed no clear survival benefit in performing hyperthermic intraperitoneal chemotherapy after cytoreductive surgery.

## 1. Introduction

Desmoplastic small round cell tumor (DSRCT), first described in 1989 [1], is a highly aggressive and very rare soft tissue sarcoma with an incidence of 0.3 per million [2]. It afflicts mostly adolescent and young adult males, who present with non-specific abdominal symptoms from multiple predominantly intra-abdominal tumors [3]. Patients often remain asymptomatic until the tumor burden is high and ascites occur as a result of peritoneal invasion, causing symptoms such as abdominal pain, distension, constipation and weight loss [4,5,6,7]. The origin of the tumor is unknown [8]. The disease is often advanced at presentation, with almost half of the patients having extra-peritoneal metastases at diagnosis [9]. Common sites of extra-peritoneal metastases are the liver, lymph nodes, and lung [8,10].

There is no consensus on the best treatment approach for DSRCT, but several studies have reported improved survival with multimodal treatment that consists of systemic therapy combined with surgery and radiotherapy [9,11,12,13,14]. Although hyperthermic intraperitoneal chemotherapy (HIPEC) has been employed after cytoreductive surgery (CRS), its benefit remains unclear. The aim of this review is to identify the role of HIPEC after CRS in patients with DSRCT. 

## 2. Workup and Staging

DSRCT is a small blue round cell tumor characterized by clusters of round or oval cells embedded in a prominent hypervascular desmoplastic stroma composed of fibroblasts or myofibroblasts [15]. Immunohistochemistry typically demonstrates multi-lineage differentiation, with variable expression of epithelial, myogenic and neural markers [16]. The molecular hallmark of DSRCT is the reciprocal chromosomal translocation t(11;22)(p13;q12), which results in the fusion of the Ewing Sarcoma (EWS) gene to the Wilms’ tumor (WT1) gene [17]. The detection of the EWSR1 rearrangement by fluorescence in situ hybridization (FISH) or the EWSR1-WT1 fusion transcript by reverse transcription-polymerase chain reaction cinches the diagnosis [18].

Cross-sectional imaging with contrasted computed tomography scans of the abdomen and pelvis reveals multiple peritoneal masses without an apparent primary organ of origin or confluent diffuse masses inseparable from the bowel [19]. The dominant mass is located in the retrovesical or rectouterine region in more than half of the patients [19]. The tumors are often described as moderately heterogeneous solid abdominal tumors with hypodense patches indicating necrosis [7,20]. Calcifications have been reported in 29% of cases [19]. Computed tomography or magnetic resonance imaging provides an idea of disease distribution and its extent, although the true extent is systematically underestimated [21]. Although diagnostic laparoscopy has been recommended in the evaluation of peritoneal metastases of other cancer types to evaluate the peritoneal cancer index (PCI) and obtain histology [22], it is not recommended in DSRCT.

Functional imaging has been explored for evaluation of treatment response since DSRCT lesions clearly demonstrate metabolic activity on fluorodeoxyglucose (FDG) positron emission tomography [19,23] and FDG uptake has shown a correlation with histopathologic tumor response [24]. One study reported a greater decrease in metabolic activity (51%) than a decrease in the size of disease (23%) after chemotherapy [23]. In addition, DSRCT is less likely to show morphologic response because of the stromal composition of the lesions [6], and these volumetric changes may not be immediately apparent after chemotherapy [19]. Fluorodeoxyglucose positron emission tomography might hence be valuable in post-treatment assessment, with the additional benefit of evaluating for distant metastases and assisting in the staging of disease [19,23,24].

The American Joint Committee on Cancer (AJCC) staging system does not account well for DSRCT in view of the unknown primary origin and multifocal nature of disease. The MD Anderson Cancer Center (MDACC) hence developed staging criteria to illustrate disease burden (Table 1), but it has yet to be validated [4,5]. These staging criteria consist of a combination of PCI, the presence or absence of liver metastases, and extra-abdominal metastases. 

The Memorial Sloan Kettering Cancer Center (MSKCC) has more recently suggested an image-based risk stratification system, based on the presence of serosal or parenchymal liver lesions and/or ascites at the time of diagnosis (Table 2) [25]. This risk stratification system assigns patients into three risk categories based on the presence of ascites and/or liver metastases at diagnosis. Based on their cohort of 130 patients, each category was assigned a 5-year overall survival (OS) estimate. The intention is for prognostication to guide treatment decisions, but in view of the overlap of confidence intervals for the high- and very high-risk categories and the lack of external validation of this system, its significance is not yet clear.

After complete diagnosis and evaluation, cases should be discussed in a multidisciplinary meeting at a specialized sarcoma center to formulate a treatment plan. Although the literature often describes DSRCT separately in children or young adults, similar treatment should be considered [26].

## 3. Induction Chemotherapy

Given the chemosensitivity of DSRCT and the high proportion of metastatic disease, initial treatment is usually systemic chemotherapy. The regimens employed include those for soft tissue sarcoma which incorporate ifosfamide and doxorubicin or those established for Ewing sarcoma which are most commonly the P6 regimen (cyclophosphamide, doxorubicin, vincristine, ifosfamide, and etoposide) [6,27] or VAIA (vincristine, dactinomycin, ifosfamide, and doxorubicin) [14]. Volumetric response to systemic chemotherapy is an indication of tumor biology and combined with the absence of extra-abdominal metastases and a low peritoneal tumor burden, aids in identifying suitable surgical candidates [10,12]. Hayes-Jordan et al. recommend at least 4 months of systemic therapy before assessing the feasibility of resection, after which treatment response reaches a plateau [4,28].

## 4. Surgery

There are no clear guidelines on patient selection for surgery in DSRCT. Almost all patients receive systemic chemotherapy prior to surgery, where progression on chemotherapy indicates poor tumor biology and precludes resection [4,10]. There is insufficient evidence at present to determine if surgical resection will benefit those who progress on first-line chemotherapy but respond to second or third-line chemotherapy regimens [12]. Patients with extra-abdominal metastases are generally excluded from resection [12,29], since it portends a higher risk of recurrence and death [21,28]. However, some have found no significant impact of extra-abdominal metastases on overall survival (OS) [13,30]. The point in the treatment trajectory when extra-abdominal metastases should be determined remains unclear: at diagnosis or after chemotherapy, as practiced in some centers [12,13]. Liver metastases are intra-abdominal lesions but are considered extra-peritoneal metastases, and consensus is lacking on its consequences. In a French study, no survival benefit of CRS over chemotherapy alone was found for patients with liver metastases. Therefore, the authors advocated for excluding patients with liver metastases from resection [9]. Conversely, the team in MDACC did not find a significant impact on OS if the liver metastases were amenable to complete resection or ablation and would consider CRS [28,29]. However, recurrence-free survival was shorter in patients with liver or portal nodal metastasis versus patients without (14 vs. 38 months, *p* = 0.02) [28]. 

**Figure 1:**
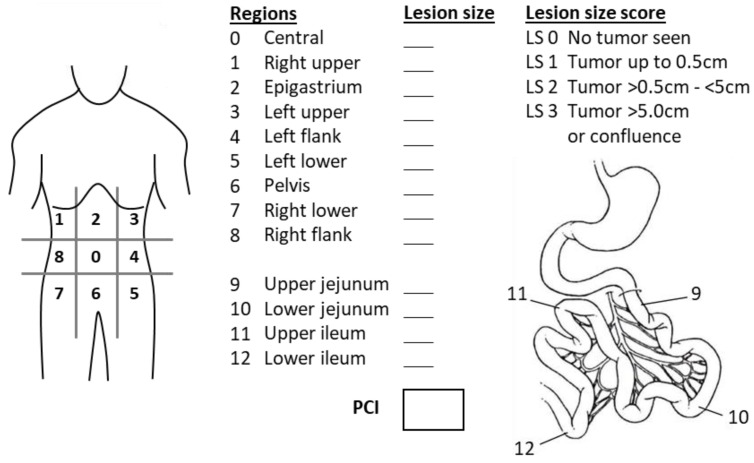
Peritoneal Cancer Index (PCI). Each region (total of 13 regions) should be scored based on the largest tumor present. All 13 scores should be added to a total Peritoneal Cancer Index (PCI), ranging from 0 to 39.

In addition to the presence of extra-abdominal and extra-peritoneal metastases, is the peritoneal tumor burden, which is assessed by the Peritoneal Cancer Index (PCI), the third major prognostic factor [31]. The PCI scoring system divides the peritoneal cavity into 13 regions, comprising nine abdominopelvic regions and four small bowel regions. In each region, a lesion size score (LS) is recorded according to the largest tumor present: LS 0) no tumor; LS 1) tumor up to 0.5 cm; LS 2) tumor between 0.5 cm to 5 cm; and LS 3) tumor > 5 cm. (Figure 1). The final PCI score is the sum of the lesion size scores of all 13 regions, ranging from 0 to 39. There is no defined maximum PCI cut-off to preclude a resection for DSRCT. In fact many studies do not report PCI scores, perhaps due to the tedious process of scoring, especially in this often advanced disease. A wide range of PCIs from 0 to 33 at diagnosis has been reported [29], but median PCIs in the literature for patients undergoing CRS ranges between 13 and 16 [10,13]. Although some have found no association between PCI and survival [28], Honoré et al. found that few patients with PCIs above 12 achieve disease-free survival (DFS) beyond 5 years [10], and Stiles et al. reported improved OS with PCI below 16 (45 vs. 32 months, *p* = 0.010) [13].

**Figure 2:**
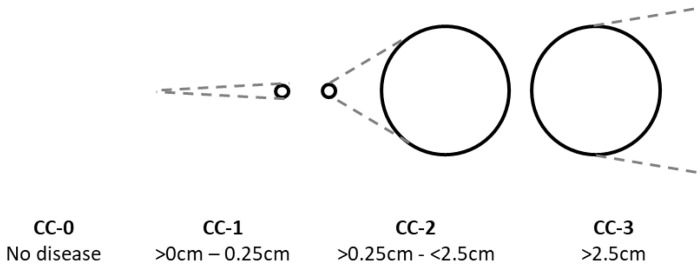
CC-score: completeness of cytoreduction after surgery.

The fourth prognostic factor is the completeness of CRS [32,33]. Complete CRS may be achieved by removing all macroscopically visible tumors by means of peritonectomy, visceral resections, or the local treatment of liver metastases, and it has been described for peritoneal surface malignancies such as pseudomyxoma peritonei, colorectal cancer, mesothelioma, ovarian cancer, and gastric cancer with a demonstrated improvement in overall survival and loco-regional control [33]. The completeness of cytoreduction (CC) score is applied post-resection as follows: CC-0) no residual macroscopic disease; CC-1) residual nodules < 2.5 mm; CC-2) residual nodules between 2.5 mm and 2.5 cm; and CC-3) residual nodules > 2.5 cm [31], see Figure 2. Complete cytoreduction is defined as CC-0 and CC-1 [31].

The ability to achieve complete CRS strongly influences prognosis and should be considered prior to undertaking a major morbid surgery. Complete CRS improves both overall and progression-free survival [9,10,21], with a median OS of 24 months with incomplete resection in contrast to 36 months after complete surgery (*p* = 0.012) [10]. In patients carefully selected for surgical exploration, 71–75% of these patients can actually undergo a complete macroscopic resection of all tumor deposits [6,10,12]. A complete resection may not be possible if the disease is located at crucial anatomic sites, for example, extensive small bowel involvement or disease at the porta hepatis [34]. When complete CRS is not feasible, debulking surgery may still confer a survival benefit. Debulking surgery is described as the removal of at least 90% of tumor deposits [5,6], and demonstrated improvement in 3-year OS from 26% without surgery to 62% after debulking (*p* = 0.031) [5]. Similarly, Lal et al. described a significant improvement in 3-year OS of 58% after debulking in contrast to 0% in patients without resection (*p* < 0.001) [6]. Debulking could also potentially prevent future complications from bulky disease or palliate abdominal symptoms [10]. 

The ultimate goal of surgery is complete cytoreduction but the potential risks of morbidity should be considered since this could prevent or delay postoperative treatments such as chemotherapy and/or radiotherapy. If the expected surgical morbidity and mortality from optimal cytoreduction are excessive, the surgeon may consider debulking surgery as an alternative.

## 5. HIPEC

In the setting of disseminated abdominal disease, surgical resection alone may not provide durable control, as microscopic residual disease appears almost inevitable. The theoretical benefit of adding HIPEC immediately after CRS is to eradicate potential microscopic disease [35]. Drains are placed in the abdominal cavity for the perfusion of high-dose heated chemotherapy, which is continuously circulated for 30 to 90 min. This can be achieved via an open coliseum or closed technique. The plasma–peritoneal barrier allows the locoregional delivery of high-dose chemotherapy with low systemic concentrations, reducing the adverse effects associated with chemotherapy [33]. Tissue penetration ranges between 1–3 mm and depends on the drug, temperature and duration of intraperitoneal chemotherapy [36]. Hyperthermia theoretically increases cytotoxicity, may enhance the efficacy of certain drugs, and increase the penetration depth of chemotherapy into tissues [37]. HIPEC has been proposed for a variety of diseases including pseudomyxoma peritonei, mesothelioma, colorectal, ovarian, and gastric peritoneal metastases [38,39,40,41]. However, the available literature is conflicting, and few randomized trials of CRS with HIPEC versus CRS alone have been performed. Many studies were either underpowered or terminated prematurely.

The first report of CRS with HIPEC in DSRCT was in 2004 [42] and subsequently in 2007 for the pediatric population [43]. Adding to the challenge of treating a rare disease with frequent extra-peritoneal metastases precluding resection, another difficulty presents in the wide practice variation of utilized chemotherapy agents, dosing, carrier solution, level of hyperthermia, and HIPEC duration [33]. The most common HIPEC regimen involves cisplatin 100 mg/m^2^ at 41°C for 90 minutes, as seen in Table 3. Cisplatin is an alkylating agent commonly used in ovarian cancer and mesothelioma, but its pharmacokinetic profile is less favorable compared to other intraperitoneal chemotherapeutic agents [35]. The distribution of DSRCT is mainly serosal with diffuse involvement of the peritoneal cavity similar to ovarian/primary peritoneal cancer and mesothelioma, hence intraperitoneal cisplatin has been utilized since it was first reported by Gil et al. in 1992 [42]. Table 3 summarizes the HIPEC regimens and treatment details for DSRCT described in the literature thus far.

At present, there is no clear evidence on the benefit of performing HIPEC after CRS for soft tissue sarcoma with peritoneal metastases. A meta-analysis of CRS with HIPEC in peritoneal sarcomatosis from a range of histologies including DSRCT reported a pooled median OS of 29.3 months for CRS with HIPEC, compared to a median OS of 13–18 months in patients treated with CRS, chemotherapy and radiotherapy [50]. The authors concluded that HIPEC may improve outcomes in some patients with peritoneal metastases from soft tissue sarcoma, but the level of evidence remains poor as the quality of the included studies was low [50]. Specifically for DSRCT, a phase 2 trial comprising 20 patients undergoing CRS and oxaliplatin HIPEC at MDACC achieved a 3-year OS of 79% with a median OS of 58.4 months from diagnosis [28]. Despite the improvement in survival, the latest study by the same group failed to demonstrate a statistically significant impact on survival with the addition of HIPEC after complete CRS [12]. Correspondingly, a collaborative nationwide study by the French networks found no significant difference in survival after incorporating intraperitoneal chemotherapy to complete CRS, but described a 30% increase in complication rate [48].

The rates of grade III–IV morbidity after CRS with HIPEC in all peritoneal surface malignancies range between 22 and 34% and 30-day mortality rates are 0.8–4.1% [51]. A meta-analysis specific for CRS with HIPEC in peritoneal metastases from soft tissue sarcoma found an incidence of 17.4% for complications requiring invasive intervention [50]. In addition, the intraperitoneal chemotherapy agent utilized may carry specific risks. Cisplatin induces renal tubular damage, with a reported 5.4% incidence of nephrotoxicity which can progress to chronic renal failure necessitating dialysis [52]. This may be prevented with adequate hydration and the use of intravenous sodium thiosulfate during HIPEC [53]. Hematologic systemic toxicity rates of 5.3% have also been described with cisplatin HIPEC, namely bone marrow suppression with mild leukopenia [54]. The addition of HIPEC to CRS in DSRCT is associated with significantly higher postoperative morbidity of 40% compared to 10% in surgery alone, although the mortality rate was not different [48].

The morbidity of a multimodal treatment approach may be cumulative. The combination of neoadjuvant chemotherapy, CRS with HIPEC and whole abdominopelvic radiation therapy (WART) resulted in 84% of patients experiencing grade 3 or higher toxicities [55]. Treatment complications may not be limited to the perioperative period, with gastroparesis, adhesive bowel obstruction, and hemorrhagic cystitis reported one year or more after CRS with HIPEC [13]. These patients required long-term parenteral nutrition, hospitalizations and additional procedures [13].

All things considered, patients contemplated for CRS with or without HIPEC should have excellent performance status, good cardiovascular health, and no liver or renal dysfunction [28]. Satisfactory renal function is especially crucial in view of potential nephrotoxicity with cisplatin HIPEC [52]. The PCI in the literature for HIPEC in DSRCT ranges between 0 and 33 [5,13,29,45], with no PCI cutoff established for either CRS or HIPEC. The prerequisite for HIPEC is complete cytoreduction, as the depth of penetration of intraperitoneal chemotherapy is limited, for example only 1–3 mm for Cisplatin [36]. Median overall survival after CRS with HIPEC in DSRCT is 63.1 months for patients achieving a CC-2 or better resection (residual disease < 2.5 cm), in contrast to 26.7 months for patients undergoing a CC-3 resection [29]. As its benefit has not been established, HIPEC is not a uniform procedure after CRS in DSRCT and is performed at each unit’s discretion. The available literature describes 23–72% of DSCRT patients receiving HIPEC after a complete CRS [12,48].

## 6. Postoperative Consolidative Treatment

Considering the high risk of relapse, postoperative treatment is critical. After surgery, further consolidative regimens with radiation therapy and systemic therapy have been employed to target microscopic disease. Whole abdominopelvic radiotherapy is delivered in view of the diffuse peritoneal involvement [32] and recently intensity-modulated radiation therapy has been used to reduce the gastrointestinal and hematologic toxicities associated with radiation therapy [56]. A study by the French Sarcoma Group evaluated adjuvant radiation therapy after cytoreductive surgery and showed improved three-year overall survival from 37.6% to 61.2% (*p* = 0.045) and improved peritoneal progression-free survival (*p* = 0.006) [57]. In addition, Honoré et al. have identified postoperative whole abdominopelvic radiotherapy to be prognostic for patients achieving a disease free interval of at least 5 years [10]. However, despite the potential benefits for survival and disease control, this multimodality approach including WART is associated with high toxicity rates [55,58]. The benefit of adjuvant chemotherapy in DSRCT is still not known. Systemic therapies are being explored for DSRCT refractory to conventional therapy. These include targeted therapies such as tyrosine kinase inhibitors (e.g., pazopanib, imatinib and sorafenib), mTOR (mammalian target of rapamycin) inhibitors and anti-type-1 insulin-like growth factor receptor antibody, as well as immunotherapy [59,60,61]. They are mostly being studied in basket trials and the results are pending.

## 7. Conclusions

Aggressive multimodality treatment and improvements in therapeutics have translated to improved DSRCT patient outcomes. Currently, the results of the largest series of 187 patients with DSRCT have been published by the MDACC group. They have shown an improvement in 5-year OS from 5% before 2003 (without multimodal treatment) to a 5-year OS of 25% with multimodal treatment [12]. However, the prognosis of DSRCT remains poor as patients who respond well to initial therapy eventually recur both intraperitoneally and extraperitoneally [28]. Despite a median OS of 60 months reported after trimodality treatment (induction chemotherapy, CRS with HIPEC and WART), the median DFS was only 10 months [55]. 

Induction chemotherapy and complete cytoreduction are essential cornerstones in the treatment of DSRCT. Although CRS and even debulking surgery have demonstrated survival benefits, they should be restricted to patients who respond to chemotherapy and those without extra-peritoneal metastases. To proceed to surgery in the presence of resectable liver metastases remains debatable. If complete CRS is technically feasible, there is no proven PCI ceiling to preclude a resection but it is clear that a high PCI is a poor prognostic factor. The additional value of HIPEC, regardless of regimen, remains unclear, and in view of the associated toxicity of HIPEC without a proven survival benefit, its use should be restricted to a very selected patient population and preferably within a study context.. The combination of induction chemotherapy, surgery, and WART has been included in the latest ‘Standard of Care and Treatment Recommendations’ for pediatric non-rhabdomyosarcoma soft tissue sarcoma from the European Paediatric Soft Tissue Sarcoma Study Group (EpSSG) [62].

To increase knowledge of this rare disease and explore further the role of HIPEC, increasing patient numbers for data collection is essential. This highlights the importance of the International DSRCT registry/retrospective database, initiated by the Transatlantic Australasian Retroperitoneal Sarcoma Working Group (TARPSWG). Clear listing of inclusion criteria and details of applied therapies is needed in order to evaluate the role of HIPEC and to be able to compare between patient groups.

## Figures and Tables

**Table 1:** MD Anderson Cancer Center DSRCT staging criteria.

Stage	PCI *	Liver Metastases	Extra-Abdominal Metastases
I	<12	No	No
II	>12	No	No
III	Any PCI	Yes	No
IV	Any PCI	Yes or no	Yes

* PCI: peritoneal cancer index.

**Table 2:** Memorial Sloan Kettering Cancer Center image-based risk stratification.

Risk	Ascites	Liver Lesions	Estimated 5-Year OS * (95% CI)
Intermediate	No	No	61% (40–76%)
High	Either ascites or liver lesions		16% (6–29%)
Very high	Yes	Yes	8% (1–29%)

* OS: overall survival.

**Table 3:** Publications of HIPEC in DSRCT.

Author	Year	#	HIPEC Details	
Bexelius [44]	2021	1/1	**Criteria**	EAM: No, EPM: Yes, liver metastases, PCI: unknown, complete CRS: Yes
			**Regimen**	Cisplatin 100 mg/m^2^ (90 min, 41 °C)
			**Outcome**	No comparison made, n = 1
Campos [7]	2020	5/11	**Criteria**	Surgery/HIPEC patients not separately described, complete CRS: Yes
			**Regimen**	Cisplatin + doxorubicin (details unknown, n = 4) Doxorubicin + docetaxel (details unknown, n = 1)
			**Outcome**	No specific HIPEC outcomes
Fan [45]	2015	3/3	**Criteria**	EAM: No, EPM: No but unclear, PCI: range 4–12, complete CRS: Yes
			**Regimen**	Cisplatin (dose unknown, 90 min, 41.5 °C)
			**Outcome**	No specific HIPEC outcomes
Hayes-Jordan [5]	2010	8/8	**Criteria**	EAM: 1/8, EPM: 2/8 (liver), PCI: range 3–33, complete CRS: Yes
			**Regimen**	Cisplatin 100 or 150 mg/m^2^ (90 min, 40–41 °C, n = 7) Cisplatin 50 mg/m^2^ + mitoxantrone (details unknown, n = 1)
			**Outcome**	OS at 3 years: 71% (HIPEC) vs. 62% (debulking), *p* = 0.031 DFS at 12 months: 53% (HIPEC) vs. 14% (debulking), *p* = 0.351
Hayes-Jordan [46]	2012	13/13	**Criteria**	Details not available + mixed tumor cohort
			**Regimen**	Cisplatin 100 mg/m^2^ (90 min, 40.5 °C) + intravenous sodium thiosulfate
			**Outcome**	Details not available + mixed tumor cohort
Hayes-Jordan [29]	2014	26/26	**Criteria**	EAM/EPM: Yes, but numbers unknown, PCI: range 0–33, complete CRS: 24/26
			**Regimen**	Cisplatin 100 mg/m^2^, max 130 mg (90 min, temperature unknown)
			**Outcome**	No specific HIPEC outcomes
Hayes-Jordan [47]	2015	21/21	**Criteria**	No DSRCT specific details (mixed tumor cohort)
			**Regimen**	Cisplatin 100 mg/m^2^ (perfusion time unknown, 41 °C)
			**Outcome**	No specific HIPEC outcomes + mixed tumor cohort
Hayes-Jordan [28]	2018	14/14	**Criteria**	EAM: No, EPM: 8/14 (hepatic or portal disease), PCI: Not DSRCT specific (mixed tumor cohort), complete CRS: Yes
			**Regimen**	Cisplatin 100 mg/m^2^ (90 min, 41 °C) + intravenous sodium thiosulfate
			**Outcome**	No specific HIPEC outcomes + mixed tumor cohort
Honoré [9]	2015	2/23	**Criteria**	No HIPEC specific details
			**Regimen**	Oxaliplatin 300 mg/m^2^ + irinotecan 200 mg/m^2^ (30 min, 43 °C) + intravenous fluorouracil 400 mg/m^2^
			**Outcome**	No specific HIPEC outcomes
Honoré [48]	2017	9/48	**Criteria**	EAM/EPM: No, PCI: median 16 (HIPEC and EPIC patients), complete CRS: Yes
			**Regimen**	Cisplatin (dose unknown, 60 min, 41 °C) Cisplatin 120 mg + mitomycin C 75 mg/m^2^ (30 min, 42 °C) Cisplatin + mitomycin + irinotecan (dose and perfusion time unknown, 41 °C) Oxaliplatin 460 mg/m^2^ (30 min, 43 °C) Oxaliplatin 300 mg/m^2^ + irinotecan 200 mg/m^2^ (30 min, 43 °C)
			**Outcome**	Median PCI higher with HIPEC/EPIC (median 16) compared to CRS only (median 9), *p* = 0.05 OS: 2y 54% (with HIPEC/EPIC) vs. 74% (CRS) and 5y 0% (with HIPEC/EPIC) vs. 22% (CRS), *p* = 0.085 DFS: 2y 0% (with HIPEC/EPIC) vs. 34% (CRS) and 5y 0% (with HIPEC/EPIC) vs. 14% (CRS), *p* = 0.087 Complication rate: 40% with HIPEC/EPIC vs. 10% CRS, *p* = 0.05
Honoré [10]	2019	15/71	**Criteria**	EAM/EPM: No, PCI: not HIPEC specific, complete CRS: 14/15
			**Regimen**	Cisplatin (other details unknown) Oxaliplatin (other details unknown) Mitomycin C (other details unknown)
			**Outcome**	5-year disease-free survival not improved with addition of HIPEC/EPIC (HR 1.35, *p* = 0.65)
Stiles [13]	2020	10/10	**Criteria**	EAM: 4/10, EPM: 5 or 6/10 (liver and intra-abdominal nodal disease), PCI: range 5–20, complete CRS: 9/10
			**Regimen**	Mitomycin 40 mg (90 min, 42 °C, n = 1) Melphalan 50 mg (90 min, temperature unknown, n = 1) Cisplatin 100 mg/m^2^ (60 min, 42 °C, n = 8)
			**Outcome**	No specific HIPEC outcomes
Subbiah [12]	2018	82/114	**Criteria**	Details not available
			**Regimen**	Cisplatin 200 mg/m^2^ (other details unknown)
			**Outcome**	OS: median of 2.0 years (CRS) vs. 2.6 years (CRS with HIPEC). Survival difference does not persist beyond 3 years, *p* = 0.16
Zmora [49]	2017	1/1	**Criteria**	Details not available + mixed tumor cohort
			**Regimen**	Cisplatin 100 mg/m^2^ (90 min, 41 °C)
			**Outcome**	Extra-abdominal recurrence after 14 months, died at 21 months

#: denotes number of DSRCT patients that received HIPEC/number of patients that underwent surgery; HIPEC: heated intraperitoneal chemotherapy, EPIC: early postoperative intraperitoneal chemotherapy, EAM: extra-abdominal metastases, EPM: extra-peritoneal metastases, PCI: peritoneal cancer index. OS: overall survival, DFS: disease free survival.
